# Cellular localization of p-tau217 in brain and its association with p-tau217 plasma levels

**DOI:** 10.1186/s40478-021-01307-2

**Published:** 2022-01-06

**Authors:** Malin Wennström, Shorena Janelidze, K. Peter R. Nilsson, The Netherlands Brain Bank, Geidy E. Serrano, Thomas G. Beach, Jeffrey L. Dage, Oskar Hansson

**Affiliations:** 1 Clinical Memory Research Unit, Department of Clinical Sciences Malmö, Lund University, Inga Marie Nilssons gata 53, 214 28 Malmö, Sweden; 2 Department of Physics, Chemistry and Biology IFM, Linköping University, 581 83 Linköping, Sweden; 3 Netherlands Institute for Neuroscience, Meibergdreef 47, 1105 BA Amsterdam, the Netherlands; 4 Banner Sun Health Research Institute, Sun City, AZ USA; 5 Eli Lilly and Company, Indianapolis, IN USA; 6 Stark Neurosciences Research Institute, Indiana University School of Medicine, Indianapolis, IN USA; 7 Memory Clinic, Skåne University Hospital, Malmö, Sweden

**Keywords:** Alzheimer’s disease, Biomarker, GVB

## Abstract

Recent studies highlight phosphorylated tau (p-tau) at threonine tau 217 (p-tau217) as a new promising plasma biomarker for pathological changes implicated in Alzheimer’s disease (AD), but the specific brain pathological events related to the alteration in p-tau217 plasma levels are still largely unknown. Using immunostaining techniques of postmortem AD brain tissue, we show that p-tau217 is found in neurofibrillary tangles (NFTs) and neuropil threads that are also positive for p-tau181, 202, 202/205, 231, and 369/404. The p-tau217, but not the other five p-tau variants, was also prominently seen in vesicles structure positive for markers of granulovacuolar degeneration bodies and multi-vesicular bodies. Further, individuals with a high likelihood of AD showed significantly higher p-tau217 area fraction in 4 different brain areas (entorhinal cortex, inferior temporal gyrus, and superior frontal gyrus) compared to those with Primary age related tauopathy or other non-AD tauopathies. The p-tau217 area fraction correlated strongly with total amyloid-beta (Aβ) and NFT brain load when the whole group was analyzed. Finally, the mean p-tau217 area fraction correlated significantly with p-tau217 concentrations in antemortem collected plasma specifically in individuals with amyloid plaques and not in those without amyloid plaques. These studies highlight differences in cellular localization of different p-tau variants and suggest that plasma levels of p-tau217 reflect an accumulation of p-tau217 in presence of Aβ plaque load.

**Supplementary Information:**

The online version contains supplementary material available at 10.1186/s40478-021-01307-2.

## Introduction

Tau is a microtubule-associated protein crucial for the stabilization of the neuronal cytoskeleton. Mis-localization, aggregation, and hyperphosphorylation of the protein are thought to lead to pathological tau spread and neuronal death. This scenario occurs in several different neurodegenerative disorders and aggregated phosphorylated tau (p-tau), forming neurofibrillary tangles (NFT) and neuropil threads (NT), constitutes one of the major neuropathological hallmarks of Alzheimer’s disease (AD). Importantly, phosphorylation of tau can occur at several different sites and give rise to different p-tau variants [5, 25]. Many of these variants can be detected in cerebrospinal fluid (CSF) [5]. The p-tau181 variant is the most studied p-tau biomarker and is today, together with total tau and amyloid-beta (Aβ) 42, used in clinical practice as a biomarker to detect AD pathology in patients with cognitive symptoms [9]. However, collecting CSF involves invasive lumbar puncture and thus much effort has been put into finding blood biomarkers for AD. Indeed, advances in the identification and validation of plasma biomarkers have recently led to several promising findings [9]. One of those findings involves the tau variant phosphorylated at Thr217 (p-tau217) [11]. Plasma levels of p-tau217 have been shown to associate with brain tau pathology specifically found in the presence of Aβ plaques in postmortem tissue [16, 19], which is in agreement with imaging studies demonstrating correlations between plasma p-tau217 and tau- positron emission tomography (PET) in patients with AD, but not in non-AD tauopathies [10, 19, 22]. Even more intriguing are reports showing differences in plasma p-tau isoforms in their correlation with tau PET or diagnostic accuracy. These findings indicate that the phosphorylation of tau at Thr217 might be related to a subtle and unique aspect of pathology that is different from the other isoforms.What drives this rise in plasma p-tau217 in AD and its underlying cellular pathological mechanism remains to be investigated. In the current study, we initiate such an investigation by exploring the cellular localization of p-tau217, in comparison to five other p-tau variants (p-tau181, 231, 202, 202/205, and 369/404), in the Cornu Ammonis 1 (CA1) of the hippocampus of AD patients. We also analyzed the presence of p-tau217 in 4 different brain areas (CA1, entorhinal cortex (EC) inferior temporal cortex (ITG), and superior frontal cortex (SFG)) of neuropathologically diagnosed individuals. Finally, we determined whether the p-tau217 load in these brain areas correlates with p-tau217 concentrations in antemortem collected plasma.

## Material and methods

### Individuals included in the study: Cohort 1 and Cohort 2

Samples from two cohorts (Cohort 1 and 2) were analyzed in the study. The samples included postmortem collected brain samples containing CA1, EC (Cohort 1 and 2), ITG and SFG (Cohort 2), and plasma collected near the end of life (Cohort 2). Cohort 1 (n = 23) consisted of donors from the Netherlands Brain Bank (NBB), and included non‐demented controls (NC; n = 12) and clinically diagnosed AD patients (n = 11). The demographics of the 2 groups are shown in Table 1. The presence of NFT and NT (visualized by AT8 staining) in this cohort were scored according to Braak stages I-VI [7] and the Aβ plaques (visualized by BETA-A4 staining) were scored into O, A, B, C, where O = zero, A = some, B = moderate and C = many [7]. Cohort 2 included (n = 44) participants from an antemortem-postmortem study, the Arizona Study of Aging and Neurodegenerative Disorders and Brain and Body Donation Program at Banner Sun Health Research Insititute [6]. The cohort was scored according to National Institute on Aging and Reagan Institute (NIA-RI) criteria [1], based on NFT Braak stages (I-VI) [7] and Consortium to Establish a Registry for Alzheimer Disease (CERAD) neuritic Aβ-plaque scores [17] as described earlier [16, 19]. The cohort consisted of (n = 12) individuals with Primary age related tauopathy (PART) (Braak III-IV, zero-sparse neuritic plaques), (n = 5) patients with either progressive supranuclear palsy (PSP) (n = 3) or corticobasal degeneration (CBD) (n = 2) (Braak III-IV, zero neuritic plaques meeting neuropathological criteria for CBD or PSP) (called Non-AD tauopathy), (n = 16) individuals with an intermediate likelihood of Alzheimer’s disease (Braak stage III-IV, moderate-frequent neuritic plaques) (called intermediate AD) and (n = 11) individuals with high likelihood of Alzheimer’s disease (Braak stage V-VI, frequent neuritic Aβ-plaque) (called high AD). Demographics of the 4 groups are shown in Table 2. Procedures for the collection of plasma and brain tissue from Cohort 2 have been described earlier [16, 19]. In addition, Aβ plaque and NFT/NT (visualized by Campbell-Switzer and Gallyas silver staining, respectively) load in the entorhinal cortex, hippocampus, temporal lobe, parietal lobe, and frontal lobe of Cohort 2 was scored (0–3 per region) and summarized as total plaque load and NFT/NT load (0–15) (for detailed procedures see [19]). Informed consent for the use of brain tissue, plasma, and clinical data for research purposes was obtained from all subjects or their legal representatives in accordance with the International Declaration of Helsinki [28].

**Table 1:** Demographic data and neuropsychiatric/-pathological evaluation of individuals included in cohort 2

	PART (n = 12)	non-AD tauopat. (n = 5)	AD_intermediate_ (n = 16)	AD_high_ (n = 11)
Age (years)	84 ± 9	82 ± 8	85 ± 7	83 ± 6
Females (%)	67	20	50	9
APOE 4 (%)	17	0	50	73
MMSE (scores)	25 ± 7	22 ± 6	23 ± 5	17 ± 6

Data are presented as mean ± SD or in percentage

**Table 2:** Demographic data of individuals included in cohort 2

Total (n = 23)	NC_−Aβ_ (n = 6)	NC_+Aβ_ (n = 6)	AD_intermediate_ (n = 5)	AD_high_ (n = 6)
Age (years)	72 ± 14	80 ± 9	81 ± 10	76 ± 10
Females (%)	33	0	80	17

Data are presented as mean ± SD or in percentage

### Brain sample preparation

The brain samples were post‐fixed in paraformaldehyde (PFA) (4%) for either 14–20 h (Cohort 1) or 36–72 h (Cohort 2) directly after autopsy and thereafter incubated in either phosphate-buffered saline (PBS) with 30% sucrose (Cohort 1) or in 2% dimethyl sulfoxide/20% glycerol (Cohort 2). The tissue was then sectioned in 40 μm free-floating sections and stored in cryoprotectant at − 20 °C (Cohort 1) or at room temperature (Cohort 2) until used for immunostaining.

### Immunostaining

For analysis of the cellular localization of p-tau217, hippocampal sections from AD patients in Cohort 1 were stained with antibodies directed against p-tau217 together with antibodies against p-tau 181, 231, 202/205, 202, 369/404, CD63, and Ckid (clones, source, and species shown in Table 3) as well as together with the thiophene-based ligand p-FTAA (a ligand that binds to aggregated tau and has been shown to co-label neurons immunostained for AT8 [3]). The sections were incubated for 1 h with blocking solution (BS) containing 5% goat serum (Jackson Immunoresearch) and 0.25% Triton in KPBS and then incubated with primary antibodies in BS overnight (ON) at 4 °C. The next day the sections were washed and incubated with secondary antibodies (Dylight 546 goat-anti-rabbit and Alexa 488 goat-anti-mouse (Thermo Fischer Scientific)) in BS ON at 4 °C. Double staining against p-tau217 and tau, p-tau231, GFAP (astrocyte marker), or iba-1 (microglia marker) was performed in a sequential manner, where the staining against p-tau 231, GFAP, and Iba-1 staining was performed first, followed by an incubated ON at 4 °C with a biotinylated version of the p-tau217 antibody in BS. Next day the sections were incubated in streptavidin 549 (Thermo Fischer Scientific) for 2 h. The staining against p-tau217 together with the bioligands p-FTAA was also performed sequentially, where p-tau217 was stained first followed by an incubation with p-FTAA in KPBS for 1 h in room temperature (RT). All stained sections were incubated in Sudan Black (1% in 70% ethanol) (Sigma-Aldrich) for 5 min before they were mounted with Vectashield Set mounting medium containing DAPI (Vector Laboratories). Associations between stained markers were analyzed using confocal microscopy (Zeiss LCM 800). To analyze the number of p-tau217-positive NFT and clusters with granulovacuolar degeneration bodies (GVB), three images ((3 × 0.15mm^2^) of the CA1 region was captured in two–three sections from each individual (in total 6–9 pictures) using an Olympus AX70 light microscope with the 20 × objective. Only clusters containing more than 10 GVBs were included in the analysis. The GVB clusters and NFTs in each picture of CA1 were counted manually by a blinded observer. The numbers were then averaged and presented as mean numbers of p-tau217 positive NFT/mm^2^ and p-tau217 positive GVB/mm^2^.

**Table 3:** Antibodies used in the study

Target	Antibody	Isotype	Source
p-tau217	IBA413	Rabbit IgG	Eli Lilly
p-tau217	IBA493-biotin	Rabbit IgG	Eli Lilly
p-tau181	AT270	Mouse IgG	Thermo Fisher Scientific
p-tau202/205	AT8	Mouse IgG	Thermo Fisher Scientific
p-tau231	EPR2488	Rabbit IgG	Abcam
p-tau202	CP13	Mouse IgG	Dr Peter Davies
p-tau369/404	PHF1	Mouse IgG	Dr Peter Davies
CD63	MEM-259	Mouse IgG	Thermo Fisher Scientific
Tau	A0024	Rabbit IgG	Dako
Ckid	128a	Mouse IgG	Eli Lilly
GFAP	6F-2	Rabbit IgG	Dako
Iba-1	Iba-1	Rabbit IgG	Wako

### Analysis of p-tau217 area fraction

Analysis of the p-tau217 immuno-stained area fraction in Cohort 1 and 2 was performed by acquiring three images (3 × 0.15mm^2^) of the CA1 (Cohort 1 and 2), EC, (ITG), and SFG region (Cohort 2). The 4 different brain areas were defined based on area-characteristic landmarks and pictures within each brain area were captured by randomly selecting an area in the blue channel (DAPI). Two–three sections from each individual (in total 6–9 pictures) were analyzed using imageJ and the averaged values are presented as mean OD Area fraction (%).

### Analysis of p-tau217 levels in plasma

The concentrations of plasma p-tau217 (cohort 2) were measured using the Meso scale discovery (MSD) platform as previously described [19]. In short, small-spot streptavidin-plates were blocked with PBS + 3% BSA + 2% PEG for 1 h in RT and then incubated with biotinylated IBA493 (diluted at 0.5 µg/ml in PBS + 0.1% BSA + 0.05% Tween 20 + 2% PEG) for 1 h in RT. The samples were diluted 1:2 in assay buffer (50 mM HEPES, 60 mM NaCl, 5 mM EDTA, 5 mM EGTA, 1% Triton X‐100, 1% MSD blocker A, 2% PEG) with the addition of heterophilic blocking reagent 1 (Scantibodies Inc) to a concentration of 200 μg/ml. Diluted samples were thereafter added to the wells in duplicates and left for incubation for 2 h at RT, followed by incubation with SULFO-tagged 4G10‐E2 detection antibody (diluted in the assay buffer) for and an additional 1 h in RT. The plate was read on MSD SQ120 within 5 min after the addition of 2 × MSD Read Buffer. All plate incubations were performed with shaking on a plate shaker. The lower limit of detection of the plasma P-tau217 assay was 0.48 pg/mL. Plasma p-tau217 values below the lower detection limit (14%) of the assay were imputed to the lowest interpolated value.

### Statistical analysis

Statistical analysis was performed using SPSS software (version 24 for Mac, SPSS Inc., Chicago, IL). Normal distribution was analyzed using Kolmogorov–Smirnov test. Differences between groups in Cohort 1 and Cohort 2 were analyzed using Kruskal Wallis Test corrected for Benjamini–Hochberg False discovery rate (FDR). Mann–Whitney Test was used when analyzing differences between two groups in Cohort 2. Correlation analysis was performed by Spearman correlation test. Results are presented as means ± standard deviations, and a value of *p* < 0.05 was considered statistically significant.

## Results

### Cellular localization of p-tau217

**Fig. 1:**
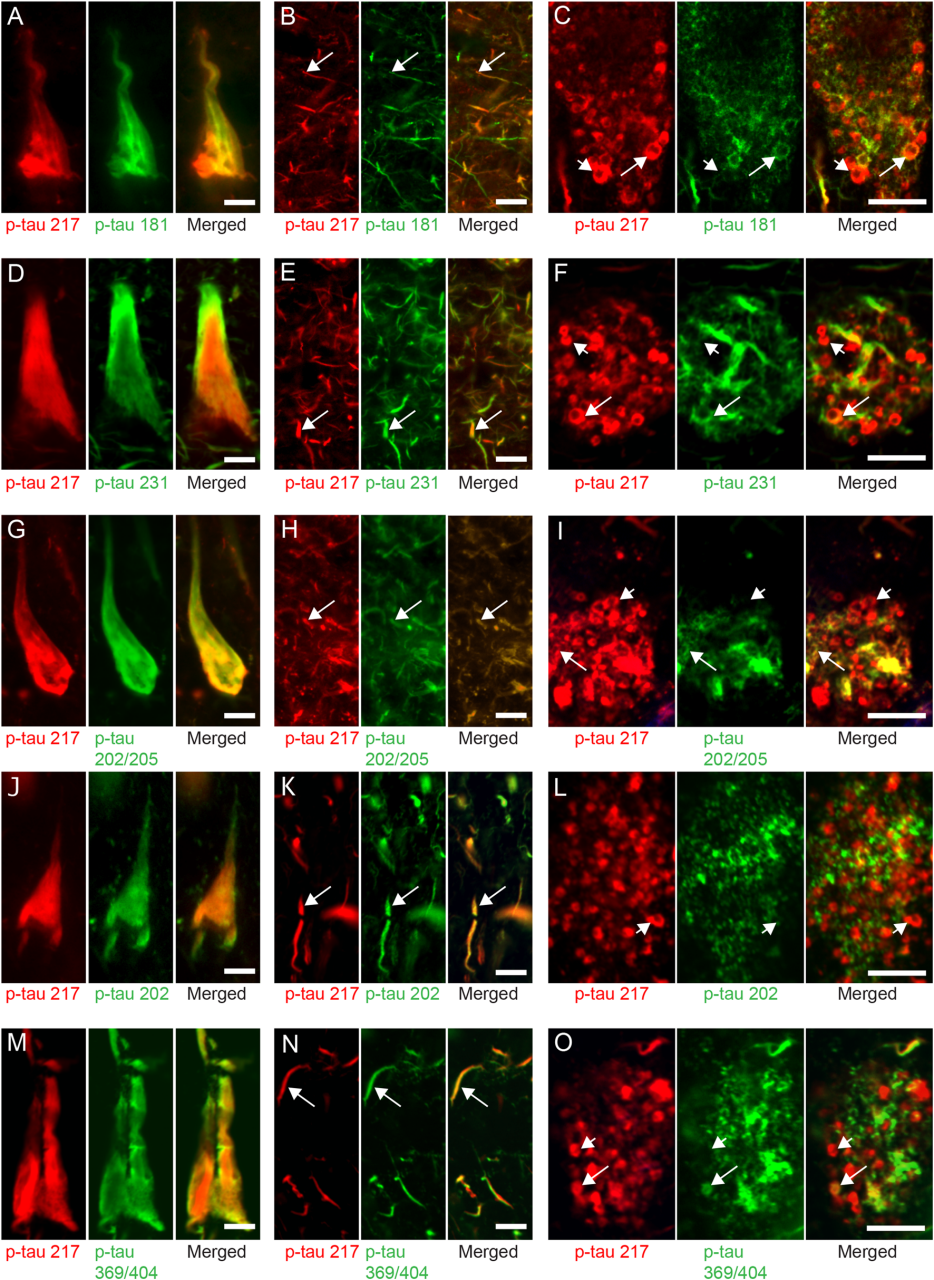
Co-immunofluorescent staining against p-tau217 and p-tau 181, p-tau 231, p-tau 202/205, p-tau 202 and p-tau 369/404. Images represent immunostaining of CA1 of an AD patient. Images in (**A**–**O**) show that p-tau217 (in red) is found in tangles, neuropil threads and in vesicle-like structures. Images in (**A**, **D**, **G**, **J** and **M**) show associations between tangles positive for p-tau217 (red) and p-tau181 (green in **A**), p-tau 231 (green in **D**) and p-tau 202/205 (green in **G**), p-tau 202 (green in **J**) and p-tau 369/404 (green in **M**). Images in (**B**, **E**, **H**, **K** and **N**) show that most neuropil threads positive for p-tau217 (red) also are positive for p-tau 181 (green in **B**), p-tau 231 (green in **E**) and p-tau 202/205 (green in **H**), p-tau 202 (green in **K**) and p-tau 369/404 (green in **N**) (indicated with longer arrow). Vesicle-like structures were clearly visualized in the p-tau217 staining (red) (arrows in in **C**, **F**, **I**, **L** and **O**), but most of them were not associated with the other five p-tau variants (indicated with short arrow) and only a few vesicles were also positive for p-tau 181 (green in **C**), p-tau 231 (green in **F**) and p-tau 202/205 (green in **G**) and p-tau 369/404 (green in **M**) (indicated with long arrow). Scalebar = 15 µm

**Fig. 2:**
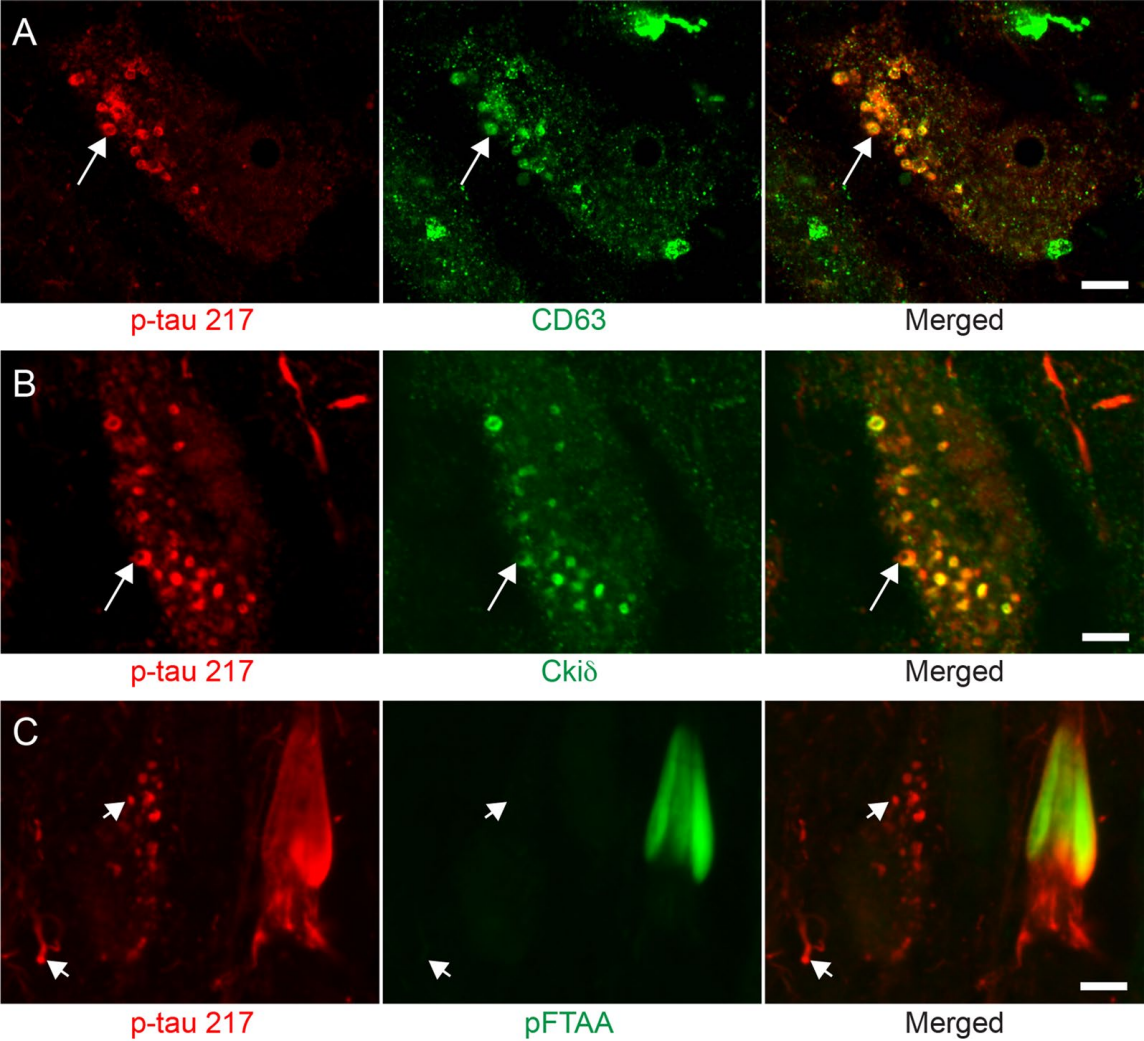
Co-immunofluorescent staining against p-tau217 and CD63, Ckid and p-FTAA. Images in (**A **and **B**) show a colocalization (indicated by arrow) between p-tau217 (red in **A **and **B**) and CD63 (green in **A**) and Ckid (green in **B**). Images in (**C**) show that p-FTAA colocalize with p-tau217 positive tangles, but not with p-tau217 positive neuropil threads and vesicles (indicated with short arrow). Scalebar = 10 µm

To investigate the cellular localization of p-tau217 in relation to other markers, we immunostained hippocampal sections from AD patients and analyzed the CA1, a brain area known to show early tau pathology in AD. The p-tau217 staining yielded classical NFT and NT, but also clusters of vesicle-like structures (Fig. 1). Co-staining showed that tangles positive for p-tau217 were also positive for p-tau 181, 231, 202/205, 202, and 369/404 (Fig. 1A, D, G, J, M, respectively), but a few NFTs positive for tau, p-tau 369/404 and p-FTAA were not positive for p-tau217 (Additional file 1: Figure S1). Similarly, most NTs positive for p-tau217 were also positive for the five other p-tau variants (Fig. 1B, E, H, K, N). Importantly, staining against p-tau217 also yielded extensive vesicle-like patterns. Although a few of these p-tau217 positive vesicles were associated with vesicles positive for p-tau181, 231, 202/205, and 369/404 (indicated by the long arrow in Fig. 1C, F, I, O, respectively), most p-tau217 positive vesicles did not (indicated by the short arrow in Fig. 1C, F, I, L, O). The vesicle-like pattern also did not associate with markers against astrocytes or microglia (Additional file 1: Figure S1). The p-tau217 positive vesicles had a diameter ranging between 0.30 and 4.13 µm, which corresponds to large endosomes such as multivesicular bodies (MVB) [2]. Indeed, a co-staining against p-tau217 and the MVB marker CD63 showed a strong association between the two markers (Fig. 2A). Moreover, since the vesicles were distributed in a pattern resembling granulovacuolar degeneration bodies (GVB), we also stained against p-tau217 together with the GVB marker Ckid [8]. This staining showed that the majority of the p-tau217 positive vesicles were also positive for Ckid (Fig. 2B) and since they, in similarity to GVBs, were found in a much higher degree in CA1 compared to for example EC (Additional file 2: Figure S2), we will hereafter call these structures GVB. Co-labelling p-tau217 together with p-FTAA further showed that p-tau217 positive tangles were positive for p-FTAA, but none of the p-tau217 positive vesicles were associated with p-FTAA (Fig. 2C).

### Presence of brain p-tau217 in neuropathological evaluated individuals in Cohort 1

**Fig. 3:**
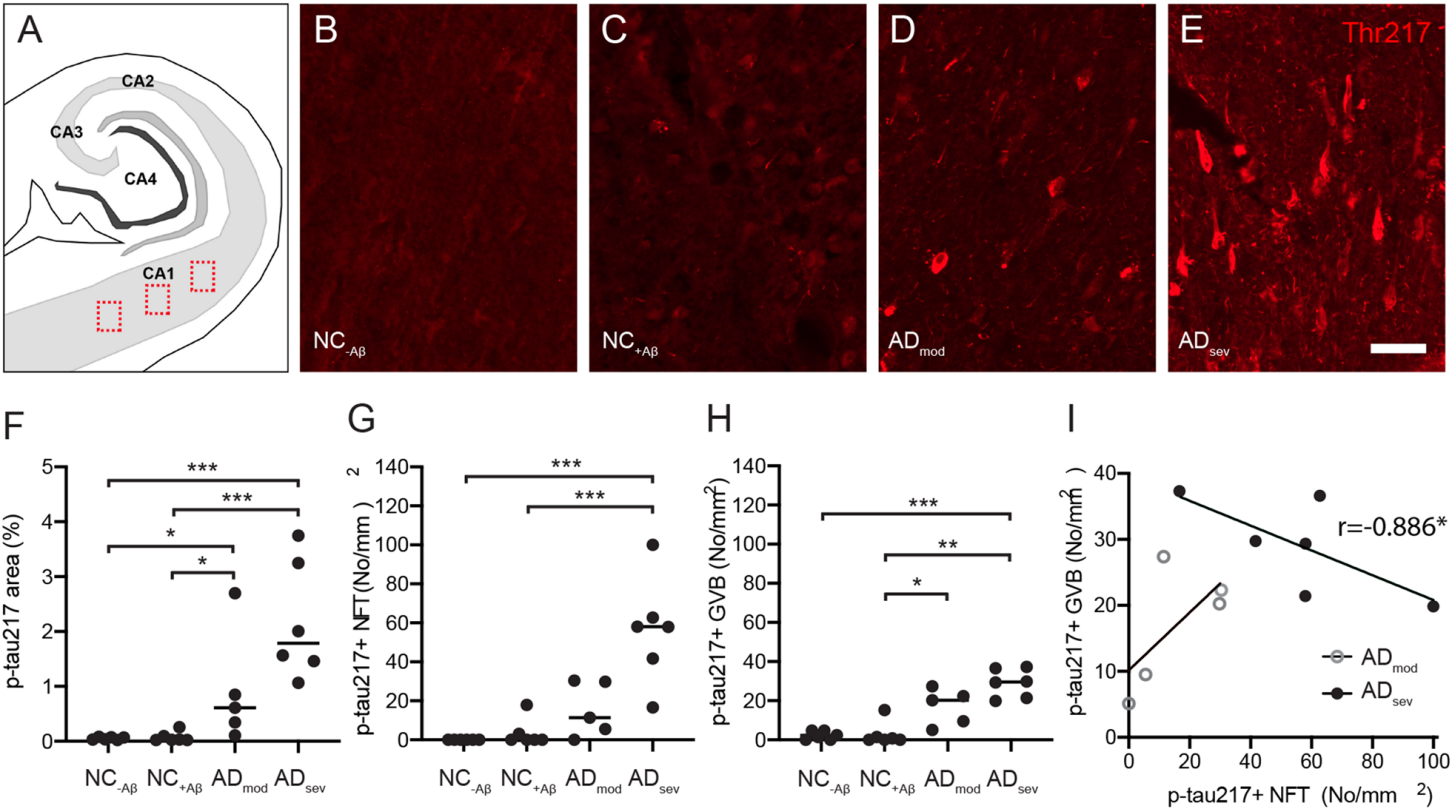
Analysis of p-tau217 area fraction, tangles and GVB in Cohort 1. Picture in (**A**) illustrates hippocampus and the CA1 area where the pictures (red dotted squares) were captured. Images in (**B**–**C**) represents pictures captured from non-demented individuals (NC) without amyloid beta (NC_−Aβ_) (**B**), NC with amyloid beta (NC_+Aβ_) (**C**), Alzheimer’s disease (AD) patients with moderate AD pathology (AD_mod_) (D) and AD patients with severe AD pathology (AD_sev_) (E). Scalebar = 40 µm. Graph in (**F**–**G**) illustrate the area fraction of p-tau217 (**F**), number of p-tau217 positive NFTs (**G**) and p-tau217 positive GVBs (**H**) in NC_−Aβ_, NC_+Aβ_, AD_mod_ and AD_sev_ All variables were significantly higher in AD_sev_ compared to NC_−Aβ_, NC_+Aβ_. Data was analyzed using was analyzed using Kruskal Wallis Test corrected for Benjamini–Hochberg False discovery rate (FDR). Scatter plot in (**I**) show that number of tangles tends to increase along with GVB in AD_mod_, whereas the opposite pattern is seen in AD_sev.._ Data in (**I**) was analyzed using Spearman correlations test. Each point represents a mean of 3 pictures from 3 sections (in total 9) from each individual. * = *p* < 0.05, ** = *p* < 0.01, *** = *p* < 0.001

Next, we investigated whether the amount of p-tau217 positive structures i.e. NFTs, NTs, and GVBs, corresponds to the overall neuropathological evaluation of NFTs and Aβ plaques in Cohort 1. The individuals where grouped based on diagnosis (non-demented controls (NC) and AD patients) and neuropathological evaluation (presence of NFT and Aβ) into 4 groups: NC_−Aβ_ (NFT Braak stage I-II, Aβ Braak O), NC_+Aβ_ (NFT Braak stage I-II, Aβ Braak A-B)), moderate AD (NFT Braak stage III-IV, Aβ Braak A-B) and severe AD (NFT Braak stage V-VI, Aβ Braak C). The area fraction of p-tau217 in pictures captured from the CA1 (Fig. 3A) was significantly higher in severe AD compared to NC_−Aβ_ (*p* = 0.0008) and NC + Aβ (*p* = 0.0007) and in moderate AD compared to NC_−Aβ_ (*p* = 0.030) and NC_+Aβ_ (*p* = 0.027) (Fig. 3B–F). Number of p-tau217 NFTs was also significantly higher in severe AD compared to NC_−Aβ_ (*p* = 0.0002) and NC_+Aβ_ (*p* = 0.001) (Fig. 3G, H). Finally, severe AD and moderate AD showed a higher number of p-tau217 GVBs compared to NC_+Aβ_ (*p* = 0.0005 and *p* = 0.020, respectively) and the same were higher in severe AD compared to NC_−Aβ_ (*p* = 0.001) (Fig. 3H). No correlation was found between the number of NFTs and GVBs when the two AD groups were analyzed together, but when analyzed separately the number of NFTs in moderate AD increased (albeit not significantly) along with the number of GVBs (r = 0.700, *p* = 0.188), whereas the opposite was seen in severe AD (Fig. 3I).

### Presence of brain p-tau217 in neuropathologically evaluated individuals in Cohort 2

**Fig. 4:**
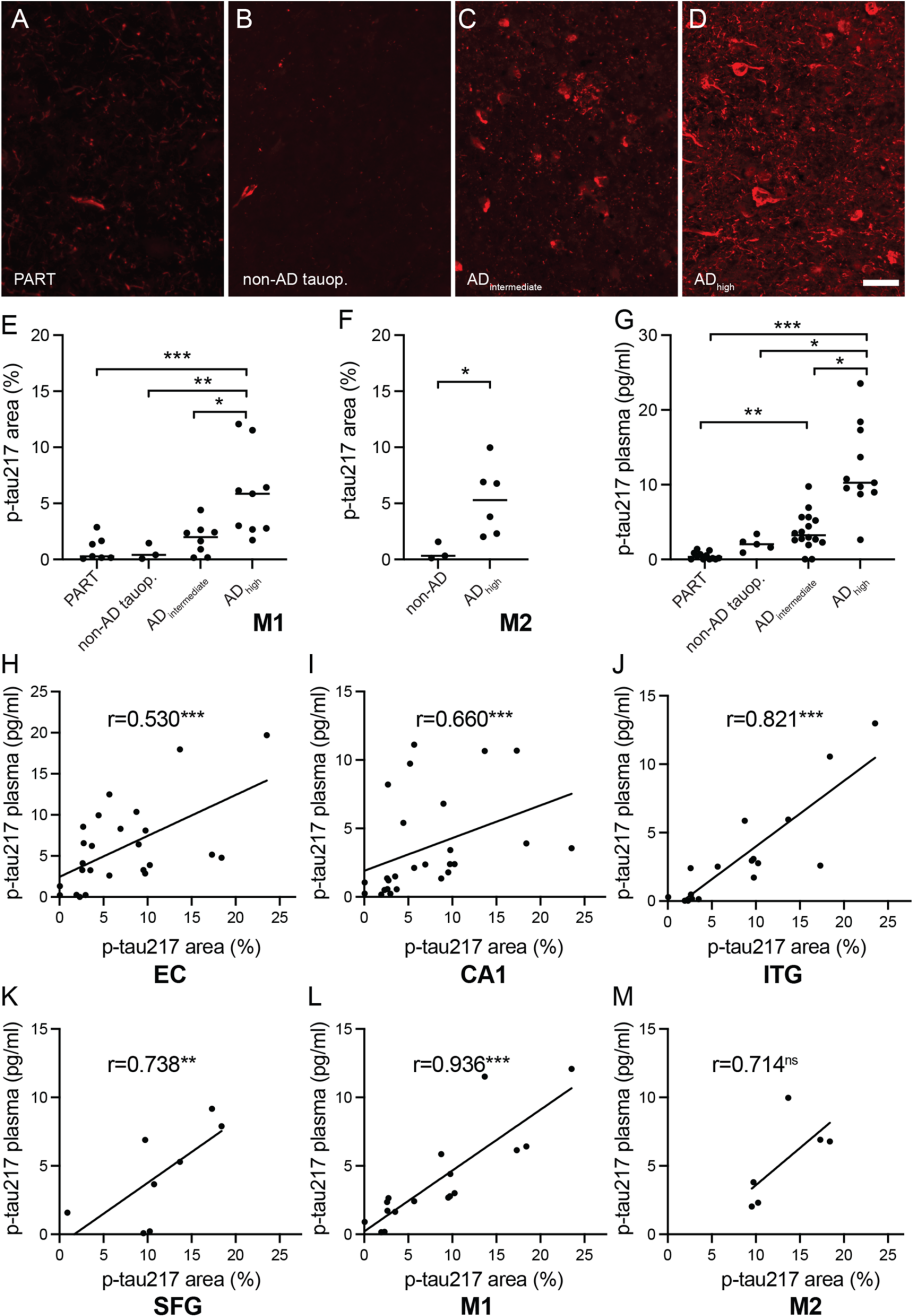
Analysis of p-tau217 in brain and plasma of Cohort 2. Images in (**A**–**D**) are representative pictures of CA1, where (**A**) shows a picture from an individual with primary age related tauopathy (PART), (**B**) from an individual with progressive supranuclear palsy (PSP) (non-AD tauop.), (**C**) from an individual with intermediate likelihood of Alzheimer’s disease (AD_intermediate_), and (**D**) from an individual with high likelihood of Alzheimer’s disease (AD_high_). Scalebar = 40 µm. Graph in (**E**) shows mean value (M1) of p-tau217 area in entorhinal cortex (EC), Cornu Ammonium 1 (CA1), inferior temporal gyrus (ITG), where AD_high_ show significantly higher p-tau217 area compared to PART and non-AD tauop. Graph in (**F**) shows mean value (M2) of p-tau217 area in EC, CA1, ITG and superior frontal gyrus (SFG), where AD_high_ show significantly higher compared to non-AD. Graph in (**G**) shows significantly higher plasma p-tau217 levels in AD_high_ compared to PART, non-AD tauop and AD_intermediate_ as well as in AD_intermediate_ compared to PART. Scatter plotts in (**H**–**M**) show significant correlations between the p-tau217 plasma values and p-tau 217 area fraction in the EC (**M**), CA1 (**N**), ITG (**O**), SFG (**P**), M1 (**Q**) and M2 (**R**) of amyloid beta positive individuals. Each point in (**E**–**M**) represents a mean of 3 pictures from 2–3 sections (in total 6–9) from each individual and data in (**E** and **G**) was analyzed using Kruskal Wallis Test corrected for Benjamini–Hochberg False discovery rate (FDR). Data in (**F**) was analyzed using Mann–Whitney Test. Data in (**H**–**M**) was analyzed using Spearman correlations test. * = *p* < 0.05, ** = *p* < 0.01, *** = *p* < 0.001

To further investigate whether the amount of p-tau217 in the brain is related to diagnosis, we analyzed the area fraction of p-tau217 in individuals of Cohort 2, which contained patients with PART, non-AD tauopathies, intermediate AD, and high AD. Four representative brain areas of each individual (number of analyzed individuals of each brain area is indicated in brackets) were analyzed: EC (n = 39), CA1 (n = 41), ITG (n = 29), and SFG (n = 12). Analysis showed that the area fraction of p-tau217 was significantly higher in high AD compared to PART in EC (*p* = 0.008), CA1 (*p* = 0.007), and ITG (*p* = 0.00008) (representative images of p-tau217 area fraction in CA1 is found in Fig. 4A–D). The p-tau217 area fraction was also significantly higher in high AD compared to non-AD tauopathies in CA1 (*p* = 0.016), and in ITG (*p* = 0.021) as well as higher in ITG of high AD compared to intermediate AD (*p* = 0.005). Further, high AD also displayed significantly higher area fraction of p-tau 217 compared to PART (*p* = 0.0008), non-AD tauopathies (*p* = 0.004) and intermediate AD (*p* = 0.018) when the mean values of EC, CA1, and ITG (M1; n = 27) were analyzed (Fig. 4E). The individuals, in which also SFG was analyzed, were grouped into high AD (n = 7) and non-AD (n = 5), and analysis showed that the p-tau217 area fraction was significantly higher in high AD compared to non-AD (*p* = 0.048). Additionally, the mean value of p-tau217 area fraction in EC, CA1, ITG, and SFG (M2; n = 9) were significantly higher in high AD compared to non-AD (Fig. 4F).

### Relations between brain and plasma p-tau217 in Cohort 2 individuals

To further investigate whether brain p-tau217 corresponds to levels of p-tau217 in plasma, we measured p-tau217 in antemortem collected plasma from the individuals in Cohort 2. The levels of plasma p-tau217 were significantly higher in high AD compared to PART (12.14 ± 5.72 vs 0.47 ± 0.48, *p* = 0.0000007, fold change 25.77), non-AD tauopathies (12.14 ± 5.72 vs 2.06 ± 0.93, *p* = 0.037, fold change 5.89) and intermediate AD (12.14 ± 5.72 vs 3.76 ± 2.50, *p* = 0.042, fold change 3.22). The latter was also significantly higher compared to PART (*p* = 0.008) (Fig. 4G). Subsequent correlation analysis of the whole cohort showed significant correlations between plasma p-tau217 and p-tau217 area fraction in all analyzed areas i.e. EC (r = 0,591, *p* = 0.000076), CA1 (r = 0.591, *p* = 0.000046), ITG (r = 0.791, *p* = 0.0000003) and SFG (r = 0.748, *p* = 0.005) as well as in M1 (r = 0.791, *p* = 0.0000009) and M2 (r = 0.900, *p* = 0.0009). When further analyzing the cohort based on the presence of amyloid plaque, we noted that the correlations in all analyzed areas were no longer significant in amyloid plaque negative individuals (*p* > 0.05) (Additional file 3: Figure S3), but remained significant in EC, CA1, ITG, SFG, and M1 of amyloid plaque positive individuals (Fig. 4H, I, J, K, L).

### Correlation analysis of p-tau217 area fraction and load of Amyloid plaque and NFT/N8 in Cohort 2

Finally, we investigated whether the p-tau217 area fraction in the different brain areas was related to the amyloid plaque load or NFT/NT load. The area fraction of p-tau217 in all 4 areas correlated with amyloid plaque load and NFT/NT load within each area (bold in Table 4), but also with both load types in most of the other analyzed areas (Table 4). EC, CA1, ITG, M1, and M2 correlated also with total amyloid plaque load and NFT/NT load, whereas SFG only correlated with total NFT/NT load (Table 4).

**Table 4:** Correlations between p-tau217 area fraction and amyloid plaque load and NFT/NT load

Brain regions p-tau217 area fraction	EC (n = 46)	CA1 (n = 48)	ITG (n = 33)	SFG (n = 14)	M1 (n = 31)	M2 (n = 10)
Total amyloid plaque (score)	0.613***	0.529**	0.676***	ns	0.805***	0.701*
Total NFT/NT (score)	0.601***	0.648***	0.754***	0.683*	0.798***	0.818**
EC amyloid plaque (score)	0.550***	0.449**	0.591***	0.636*	0.743***	0.837***
Hippocampus amyloid plaque (score)	0.586***	0.535***	0.658***	ns	0.790***	0.685*
Temporal lobe amyloid plaque (score)	0.616***	0.461**	0.661***	0.658**	0.776***	0.837***
Frontal lobe amyloid plaque (score)	0.609***	0.548***	0.669***	0.612*	0.767***	0.822**
EC NFT/NT (score)	0.532***	0.533***	0.494**	ns	0.627***	ns
Hippocampus NFT/NT (score)	0.636***	0.636***	0.599***	ns	0.825***	0.807**
Temporal lobe NFT/NT (score)	0.501***	0.549***	0.690***	0.739**	0.745***	0.914***
Frontal lobe NFT/NT (score)	0.500***	0.556***	0.734***	0.647*	0.708***	0.839**

EC = entorhinal cortex, CA1 = Cornu Ammonis 1, ITG = inferior temporal gyrus, SFG = superior frontal gyrus, M1 =  (EC + CA1 + ITG)/3, M2 = (EC + CA1 + ITG + SFG)/4, ns = not significant. Correlations was analysed using Spearman correlations test. * Indicates a significant difference at the *p* < 0.05 level. ** Indicates a significant difference at the *p* < 0.01 level. *** Indicates a significant difference at the *p* < 0.001 level

## Discussion

Our study shows that p-tau217 can be found within NFTs and NTs also containing the p-tau variants p-tau181, 231, 202, 202/205, and 369/404, a finding suggesting that phosphorylation of tau at site Thr217 often occurs at the same time as phosphorylation at other tau sites. This idea is supported, not only by previous studies demonstrating strong correlations between levels of p-tau217 and various p-tau variants in plasma and CSF [4, 10, 11, 19, 20] but also by a recently published study showing that p-tau217 can be found in the same early tangle maturation stages as many of the other p-tau variants including p-tau181, 231, 205 [18]. Our study adds to these studies by demonstrating subtle differences in the coverage of p-tau217 immunoreactivity in NFT and NT compared to the other p-tau variants. We also noted that a few tangles positive for tau, P-tau202, p-FTAA did not show p-tau217 immunoreactivity, and we thus conclude that although p-tau variants may arise at the same time during tangle maturation, they appear to accumulate in different compartments in the neurons. The most interesting finding was the persistent p-tau217 staining of vesicle-like structures in the CA1 region, something that was not seen to the same extent after staining for P-tau181, 231, 202, 202/205, and 369/404. The p-tau217 vesicle was associated with Ckid, which has been shown to efficiently distinguish intracellular GVB [8]. These structures are defined as large membrane-bound vacuoles containing aggregated proteins including p-tau [29], TDP-43 [21], and Aβ [14]. The GVBs are commonly seen in patients with tauopathies and are highly correlated with the spreading of tau pathology, in a stereotypical pattern from the entorhinal cortex to the neocortex, hypothalamus, amygdala, and eventually frontal and parietal cortex [21, 26]. Although found throughout the brain, GVBs are most frequently seen in CA1 neurons of AD patients [26]. In line with these observations, the Ckid + / p-tau217 + found in our study was most prominent in the CA1 region, but also detected in the EC, albeit in general with a smaller number of vesicles in each cell. We found no p-tau217 + vesicle structures in the ITG or SFG. However, staining of these regions was performed only on samples from Cohort 2, which were formalin postfixed for a longer period compared to Cohort 1 and may thus yield weaker staining of these structures. The function of GVBs are still not fully understood but an increasing number of studies suggest a role within autophagy, where GVB co-labeled with late (and not early) autophagy markers [8] accumulate waste awaiting intracellular degradation [8]. The formation of GVB has also been associated with necroptosis, a programmed form of neuronal necrosis characterized by the formation of necrosomes [12]. These necrosomes, which correlate in numbers with both neuronal cell loss and NFT in AD patients, co-localize with the GVB marker Ckid in CA1 [12]. Interestingly, recent studies have also found evidence pointing towards a role for GVBs in exosomal secretion, where the waist instead of being degraded is extracellularly secreted [30]. Our observed association between p-tau217 vesicles and CD63 supports this idea since this glycoprotein is a well-known marker for MVB and secreted exosomes [23]. Interestingly, seeding of tau in mice enhances the formation of GVB [27] and it has been suggested that GVB-bearing neurons show similarities to neurons in the early pre-tangles stage of tau pathology [26]. Given these findings we hypothesize that the spreading of p-tau217 through the brain can be driven by GVB formation, leading to an early and efficient secretion and propagation of the p-tau217.

One of the goals of the present study was to investigate whether the brain load of p-tau217 is related to diagnosis. For that, we analyzed 4 representative brain areas (EC, CA1, ITG, and SFG) of individuals with PART, non-AD tauopathies, an intermediate likelihood of AD, and a high likelihood of AD. Since all individuals included in the study showed tauopathy within Braak stages III-V, it was interesting to find that individuals with high likelihood of AD showed significantly higher p-tau217 area fraction compared to both PART and non-AD tauopathies. It thus appears as if phosphorylation of tau at Thr217 is an event that is somewhat selective for AD-pathology. Of note, individuals with an intermediate likelihood of AD (who just like those with a high likelihood of AD show Aβ pathology) did not significantly differ from any of the other groups suggesting that Aβ pathology can be present without significantly altered phosphorylation of tau at Thr217 in the brain.

Finally, we investigated whether plasma levels of p-tau217 reflect the load of p-tau217 in the brain. Indeed, plasma p-tau217 levels correlated with the p-tau217 area fraction in all four brain areas. The correlations were only of moderate strength, which most probably is partly due to the fact that the area fraction is a rather crude quantitative method. But it is also important to point out that p-tau217 plasma levels most probably reflect the total load of p-tau217 load in the brain and not the amount of p-tau217 in single brain areas. To account for this limitation, we also calculated the mean value of the p-tau217 area fraction of the 4 analyzed brain regions (M2). Unfortunately, only 9 individuals had values from all 4 areas, and thus we also calculated the mean value of EC, CA, and ITG (M1). The correlation between p-tau217 plasma levels and p-tau217 area in M1 increased compared to when the areas were analyzed separately and became very strong when the same correlation with M2 (i.e. all 4 brain areas) was analyzed, highlighting the idea that p-tau217 plasma levels reflect an accumulation of p-tau217 throughout the cerebral cortex. Interestingly, when we divided Cohort 2 based on the presence of Aβ pathology, we noted that the correlation between plasma and brain p-tau217 only remained in individuals with amyloid plaque loads, and the correlations within each area were improved. This finding support previous studies demonstrating a relationship between p-tau217 and Aβ pathology [15, 16], and therefore we also investigated whether the p-tau217 area fraction in the different brain regions was related to amyloid plaque/Aβ pathology. Indeed, the area fraction of p-tau217 of each brain region correlated well with both amyloid plaque loads in the total and individual brain areas, again suggesting a link between Aβ pathology and phosphorylation of tau at site Thr217.

Our study has some additional limitations, besides the ones mentioned above. First of all, antibody specificity and affinity differ, and we cannot exclude that some of our association findings (or lack thereof) are affected by such limitations. Further, the area fraction of p-tau217 analysis was performed on 4 selected brain areas and we have can thus not fully grasp the association between plasma p-tau217 and the presence of p-tau217 in the whole brain. The brain area distribution of tau lesions in PSD and CBD follows a different pattern compared to AD [13], as tau lesions a more frequently found in the frontal area and less in the hippocampal areas in the former pathologies. These differences need to be taken into account when interpreting the results, but of note also SFG of the non-AD cases (which contained n = 3 PSP, n = 1 CBD, and n = 1 PART cases) displayed lowered p-tau217 area fraction compared to high AD. Also, a recent study has shown that PART is associated with an early specific presence of AT8 positive NFT and NTs in the CA2 and less of the same in the CA1, while the opposite is seen in AD cases [24]. Such pathology-specific distribution pattern could affect our result, in particular the relationship between p-tau217 plasma levels and analysis of CA1, and may explain why the correlations are much improved when the values of all brain areas are summarized (M1 and M2). Finally, the number of individuals in different groups in Cohort 1 and Cohort 2 was rather small (ranging from 5 to 16), which may contribute to type 2 statistical errors. Hence, further studies on larger cohorts are warranted.

To conclude, our study, demonstrating the localization of p-tau217 in cellular structures specific for the p-tau variant, highlights differences between p-tau variants and invites further explorative studies aiming to understand the cellular pathways implicated in p-tau217 accumulation, seeding, and secretion. The found correlation found between p-tau217 plasma levels and p-tau217 brain load further supports the use of plasma p-tau217 as a biomarker to monitor AD pathology in future diagnostics.

## Supplementary Information

**Additional file 1: Figure S1**. Immunostaining against P-tau217 and GFAP, Iba-1, tau, p-tau 369/404 and p-FTAA. Image in (A and B) show that neither GFAP positive astrocytes (asterisks in A) nor iba-1 positive microglia (asterisks in B) is assocaited with P-tau217 positive vesicles (arrows in A and B). Images in (C-E) show that not all tangles positive for tau (C) and p-tau396/404 (D), p-FTAA (E) Scalebar = 20 µm

**Additional file 2: Figure S2**. Immunostaining against Ckid. Image in (A and B) show pictures of Cornu Ammonus 1 (CA1) and entorhinal cortex (EC) of and AD patients captured with 20 × magnification. The number of Ckid positive clusters and vesicle within each cluster (indicated with arrows) are several times higher in CA1 (A) compared to EC (B). Scalebar = 20 µm

**Additional file 3: Figure S3**. Correlation analysis between p-tau217 plasma and p-tau217 area fraction in amyloid beta negative individuals. Scatter plotts in (A-F) show how p-tau217 plasma values relates to p-tau 217 area fraction in the Entorhinal cortex (EC) (A), Cornu Ammonium 1 (CA1) (B), inferior temporal gyrus (ITG) (C), superior frontal gyrus (SFG) (D), mean value of EC, CA1 and ITG (M1) (E) and mean value of EC, CA1, ITG and SFG (M2) (F) of amyloid beta negative individuals. Each point in (A-F) represents a mean of 3 pictures from 2–3 sections (in total 6–9) from each individual and data was analyzed using Spearman correlations test.

## Data Availability

All data generated or analysed during this study are included in this published article [and its supplementary information files].
