# Supplementary figures and images for: Cellular localization of p-tau217 in brain and its association with p-tau217 plasma levels
Source: Acta Neuropathol Commun. 2022 Jan 6;10:3. doi: 10.1186/s40478-021-01307-2 (PMC8734209; doi:10.1186/s40478-021-01307-2)

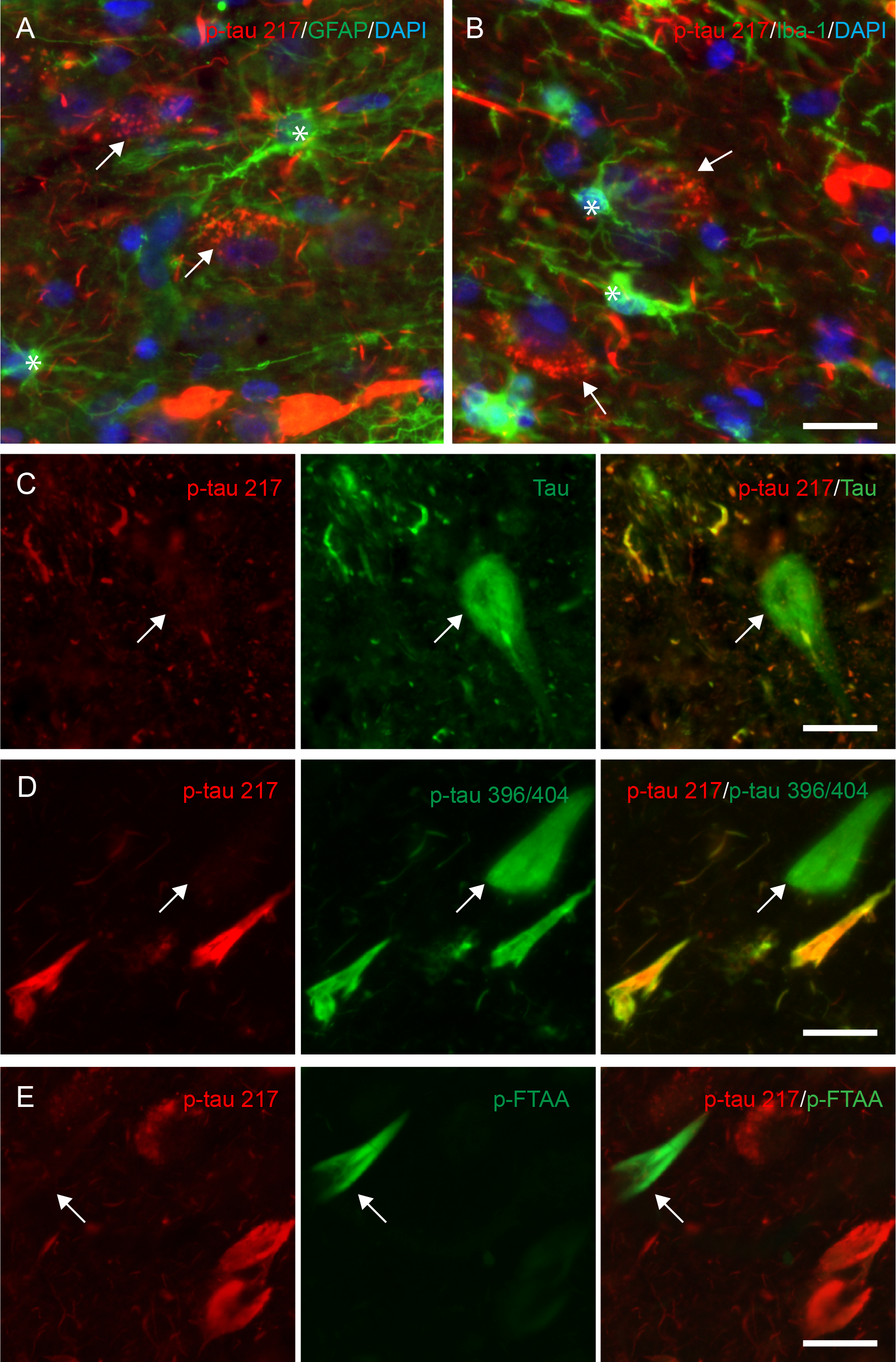

Supplement: Supplementary file 1 — Additional file 1: Figure S1. Immunostaining against P-tau217 and GFAP, Iba-1, tau, p-tau 369/404 and p-FTAA. Image in (A and B) show that neither GFAP positive astrocytes (asterisks in A) nor iba-1 positive microglia (asterisks in B) is assocaited with P-tau217 positive vesicles (arrows in A and B). Images in (C-E) show that not all tangles positive for tau (C) and p-tau396/404 (D), p-FTAA (E) Scalebar = 20 µm [file 40478_2021_1307_MOESM1_ESM.tif]

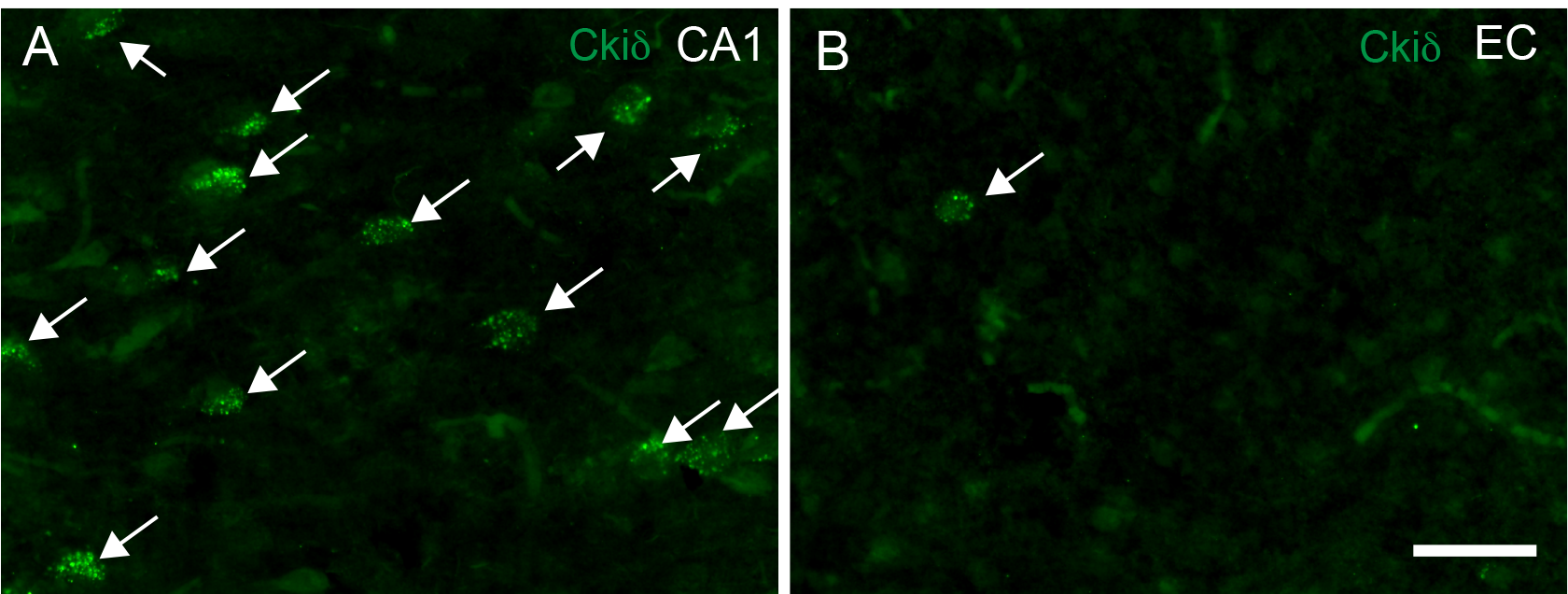

Supplement: Supplementary file 2 — Additional file 2: Figure S2. Immunostaining against Ckid. Image in (A and B) show pictures of Cornu Ammonus 1 (CA1) and entorhinal cortex (EC) of and AD patients captured with 20 × magnification. The number of Ckid positive clusters and vesicle within each cluster (indicated with arrows) are several times higher in CA1 (A) compared to EC (B). Scalebar = 20 µm [file 40478_2021_1307_MOESM2_ESM.tif]

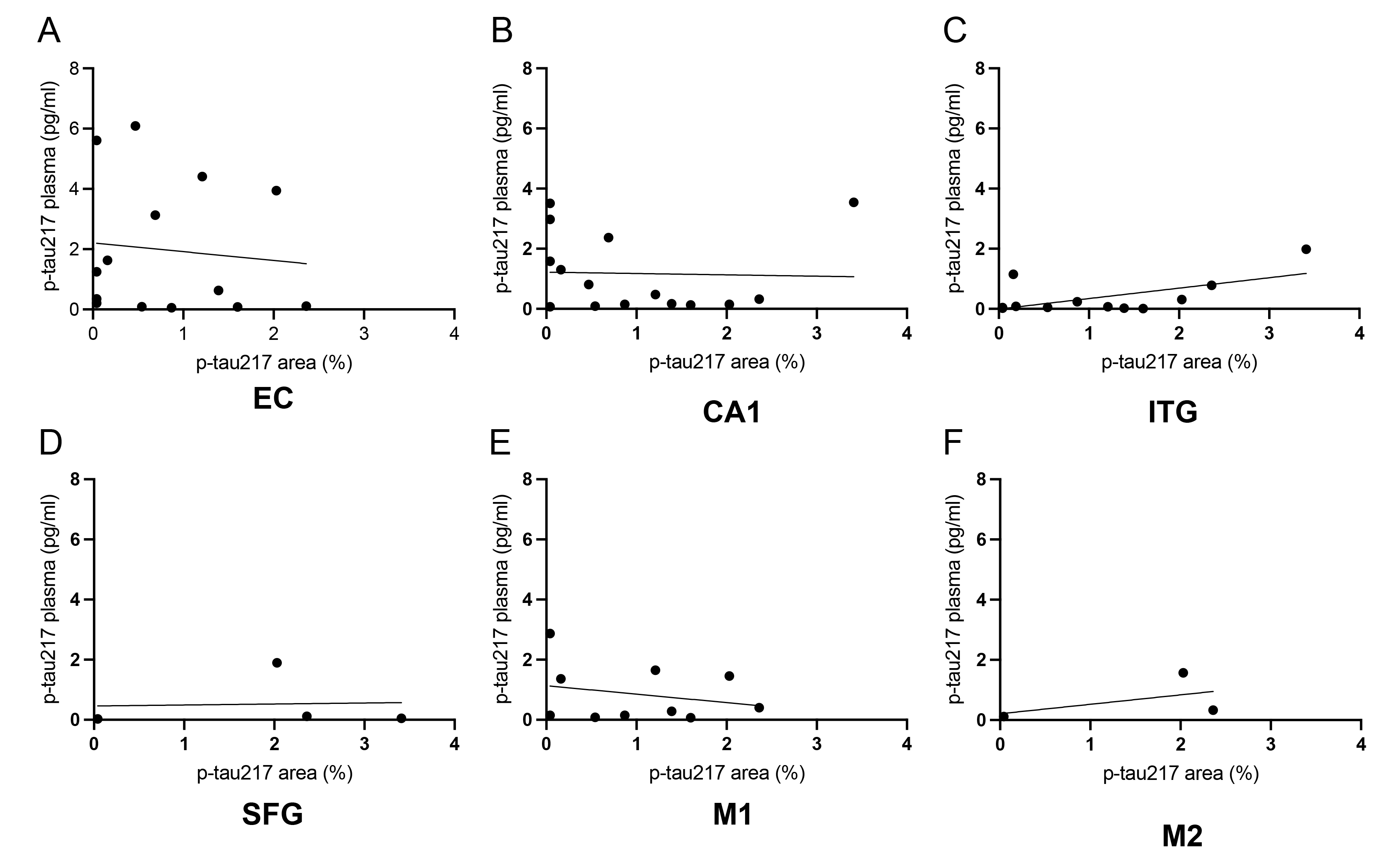

Supplement: Supplementary file 3 — Additional file 3: Figure S3. Correlation analysis between p-tau217 plasma and p-tau217 area fraction in amyloid beta negative individuals. Scatter plotts in (A-F) show how p-tau217 plasma values relates to p-tau 217 area fraction in the Entorhinal cortex (EC) (A), Cornu Ammonium 1 (CA1) (B), inferior temporal gyrus (ITG) (C), superior frontal gyrus (SFG) (D), mean value of EC, CA1 and ITG (M1) (E) and mean value of EC, CA1, ITG and SFG (M2) (F) of amyloid beta negative individuals. Each point in (A-F) represents a mean of 3 pictures from 2–3 sections (in total 6–9) from each individual and data was analyzed using Spearman correlations test. [file 40478_2021_1307_MOESM3_ESM.tif]
